# Emmett’s Operation

**Published:** 1885-08

**Authors:** 


### Emmett's Operation.

Dr. Gustav Zinke read before the American Medical Association, on "Emmett's Operation: When shall it and when shall it not be performed?"

The enthusiasm of the general profession over this operation is so unbounded that any and every cervical laceration is liable be sewed up, whether there are or are not indications therefor.

So far, enough experience has been obtained, approximately, to formulate indications for and against the operation.

Some of Dr. Zinke's conclusions are as follows:

(1) It is evident that the operation has been done unnecessarily for symptoms similar to, but other than, those arising from laceration of the cervix. Further, that it has been done imperfectly, even without preliminary treatment, in many more; and the failure to give relief, as reported by several, is due to these two causes.

(2) That from our present knowledge, we cannot, at this time, arrive at any definite conclusion, from the fact that many of the so-called consequences of laceration of the cervix uteri are not settled beyond doubt.

(3) That everyone engaged in this department should carefully select his cases, and try every known means to give relief before resource is had to operation.

(4) The operation should never be performed *eo ipso* in cases of simple fissures or lacerations of first or second degree.

(5) In cases of eversion and disease of the cervical or corporeal cavity, or both, although attended by hyperplasia and displacement, it has been observed that all symptoms abated and the parts returned to their natural condition, and that no laceration was discoverable after alleviative measures were instituted first, which alone caused the parts to return to a normal condition.

(6) There are some cases of extensive lacerations of cervix that seldom give rise to any inconvenience, and that, therefore, an operation should be deferred until symptoms arise that will call for its performance.

(7) The operation, although indicated, should never be performed until, by preparatory treatment, the parts have been brought into a healthy condition.

(8) Near, and during the climacteric period, the operation should be postponed as long as possible, and the patient not exposed to any risks, since in many cases all the symptoms subside under proper treatment, and never return on account of senile involution.

(9) The operation may be performed with perfect propriety in young women as a preventive, if the laceration is bilateral, and extends up to the cervico-vaginal junction, or beyond it, even though there are no pathological changes; indeed, it seems to be the duty of everyone who observes a lesion to that extent, to urge the operation.

(11) The operation is justifiable in any degree of laceration, and, in rare instances, even in fissures, when there exists cicatricial tissues, productive of reflex disturbances, annoying in character, and not tractable to any other treatment.

(12) The operation is absolutely indicated in all extensive tears of the os, in which the cervix is everted, its mucous membrane and the Nabothian follicles diseased, and especially if there be granular or cystic degeneration present, provided the parts have first been restored to a healthy condition by palliative treatment.

